# Feasibility and acceptability of a two-phase survey for estimating the prevalence of mental disorders in adults with type 1 diabetes

**DOI:** 10.1186/s40814-025-01669-7

**Published:** 2025-08-28

**Authors:** Jeni Baykoca, Madeleine Benton, Paul Moran, Beth Stuart, Ian Glass, Hermione Price, Khalida Ismail

**Affiliations:** 1 Research and Development, Tom Rudd Unit, Moorgreen Hospital, Southern Health NHS Foundation Trust, Southampton, UK; 2 Department of Psychological Medicine, King’s College London, London, UK; 3 Population Health Sciences Department, Centre for Academic Mental Health, Bristol Medical School, University of Bristol, Bristol, UK; 4 Wolfson Institute of Population Health, Queen Marry University of London, London, UK

**Keywords:** Type 1 diabetes, Mental disorder, Epidemiology, Mental health Long-term conditions

## Abstract

**Background:**

We assessed the feasibility and acceptability of conducting an epidemiological survey to estimate the distribution of mental disorders in a sample of adults with type 1 diabetes mellitus (T1D).

**Methods:**

Eligible participants were adults with T1D recruited from four general practices in southeast England. Phase 1 included screening measures for mental disorders in the DSM-5. Participants at phase 1 were invited to a structured clinical interview for DSM-5 at phase 2. Feasibility parameters included the proportions of those identified as eligible, consenting, and completing either or both phases, and acceptability.

**Results:**

The study population comprised 146 adults with T1D. 72% (*n* = 105) had correct contact details, were eligible and invited. 52% (*n* = 55) completed phase 1, of which 45% (*n* = 25) completed phase 2. Some measures had high rates of missing values and three mental disorders had concordant phase 1–2 pairs, namely schizophrenia spectrum and other psychotic disorders, depressive disorders, and substance-related and addictive disorders.

**Conclusions:**

Conducting a two-phase survey of mental disorders in people with T1D is feasible and acceptable and can be improved using methods to update current contact details; adding secondary care diabetes services (hospitals) as recruitment sites; reducing the screening measures; and omitting diagnostic interviews for those mental disorders already listed in routine medical records.

**Supplementary Information:**

The online version contains supplementary material available at 10.1186/s40814-025-01669-7.

## Key messages regarding feasibility

- What uncertainties existed regarding the feasibility?
  We conducted a systematic review and reported that people with type 1 diabetes mellitus (T1D) are at increased risk of nearly all mental disorders, but around half are undetected in routine care. A population-based survey of the prevalence of all mental disorders has not yet been conducted, therefore it is not possible to estimate the distribution by type of mental disorder. We therefore assessed the feasibility of conducting a two-phase survey. We adapted phase 1 to screen for all mental disorders and phase 2 to include all respondents from phase 1 to maximise the data collected and number of participants to identify which screening measures were predictive of detecting most mental disorders.
- What are the key feasibility findings?
  A two-phase population-based survey design is feasible in terms of participation rates, especially at phase 1, and acceptable for most screening measures. Acceptability could be improved by reducing the number of screening measures and omitting diagnostic interviews for those mental disorders already listed in routine medical records.
- What are the implications of the feasibility findings for the design of the main study?
  The feasibility findings highlight the need for reducing the number of phase 1 measures to include schizophrenia spectrum and other psychotic disorders, depressive disorders, anxiety disorders and substance-related and addictive disorders.

## Background

Type 1 diabetes mellitus (T1D) is a serious lifelong condition in which the pancreatic beta cells are destroyed by an autoimmune process leading to insulin deficiency and resultant hyperglycaemia. T1D constitutes around 10% of all diagnosed cases of diabetes and its incidence is increasing by 3% every year, especially in childhood [1]. T1D has a bimodal distribution of incidence with peaks in early childhood (around age 5 years) and adolescence (around age 15 years) although 50% of cases present in adulthood [2]. The aetiology remains unknown and there is no cure.

The management of T1D involves insulin self-administration with the aim of attaining optimal glycaemic control. This is critically important to minimise the risk of long-term microvascular (diabetic retinopathy, nephropathy, and neuropathy) and macrovascular complications (myocardial infarction, heart failure, and stroke) from occurring. People with T1D need to undertake daily self-care tasks, including blood-glucose and ketone monitoring, and calculating and injecting insulin doses. Despite the development of modern insulins and diabetes technologies, many people with T1D find it challenging to achieve optimal glycaemic control (typically measured by HbA1c). The reasons are multifactorial, and mental health is a significant contributing factor.

Diabetes distress can interfere with the management of T1D [3]. There is also consistent evidence that mental disorders such as depression, anxiety, and eating disorders are more common in people with T1D than the general population [4] and are associated with higher glycaemic levels, increased risk of diabetes complications and increased mortality [5]. Many prevalence studies are in highly selected and small samples. They tend to focus on one mental disorder and do not estimate its severity and its impact on self-management [6]. Despite national and international guidelines recommending mental health screening, around half of the cases of mental disorders go undetected in people with diabetes [7]. This is partly because there is no routine screening and no pathway of care for the different categories of mental disorders taking into account their severity and difficulties with self-management (as reflected by degree of hyperglycaemia, recurrent diabetic ketoacidosis (DKA) and hypoglycaemia) [8]. Among people with T1D, little is known about the prevalence and overall burden of mental disorders, including substance-related and addictive disorders, and more complex mental disorders such as personality disorders [9].

Our aims were to assess the feasibility and acceptability of undertaking a future population-based survey to estimate the distribution and severity of mental disorders in adults with T1D.

## Material and methods

### Study design

We used a two-phase survey design for its pragmatic approach to collecting unbiased population-based prevalence estimates for mental disorders, as it is not efficient or economical to conduct clinical diagnostic interviews in a large cohort of T1D [10], especially for mental disorders that are not routinely screened. In the standard two-phase survey, a screening measure for all mental disorders is used to identify potential cases in phase 1, and in phase 2 a sub-sample is randomly selected and classified by a typically more expensive gold standard clinical diagnostic interview [10]. We adapted this survey design in two ways. First, we invited all (not a random sample) respondents at phase 1 to phase 2 interviews. The reason was that as this was a feasibility study, the study sample was small, and we wanted to maximise data collection. Second, we included the screening measures for the majority of Diagnostic and Statistical Manual of Mental Disorders, 5th edition (DSM-5) mental disorders rather than one generic screening measure. This was because as we included a population of people with diabetes rather than a general population, and as based on a previous meta-analysis [7] we were anticipating higher rates of *all* mental disorders. We also did not want to risk not detecting less common mental disorders, such as autism spectrum disorder and schizophrenia spectrum and other psychotic disorders which may nevertheless have adverse effects on diabetes outcomes. We wanted to include disease specific psychological distress and it is debatable whether this can screen for mental disorders and vice versa [7]; sexual dysfunction is a hidden but common problem in the diabetes population and may not be detected in a single general screening measure [11].

### Setting

The study was set in one Primary Care Network (PCN) (a network structure where general practices are brought together on an area basis) comprising four general practices in Hampshire, England. The PCN has approximately 13,300 patients, of which there were estimated 146 adults with T1D on the PCN’s diabetes register.

### Eligibility criteria

Inclusion criteria were adults i) aged 18 years or over, ii) recorded as having a diagnosis of T1D, iii) proficient in English, iv) registered within the participating PCN, v) able to provide informed consent. Exclusion criteria were i) people younger than 18 years, ii) adults identified through the search that the general practitioner (GP) did not consent to being invited for any reason (including but not limited to, end-of-life care), iii) adults with other types of diabetes (e.g., type 2 diabetes mellitus, gestational diabetes mellitus).

### Phase 1: screening

All participants were screened for the presence of mental disorders, as well as their diabetes-related functioning. To increase participation, the survey was offered in both a paper and online format. The online survey was administered on *Qualtrics XM* [12].

The first mailing was on 10.03.2022 and participants had a 4-week period to return the survey before a reminder pack was posted. If there was no response from the reminder pack, a second reminder pack with a £10 incentive was posted after another 4 weeks. If there was no response to the third mailing after 4 weeks, a researcher (JB) phoned non-responders with an option to complete the survey over a phone call, receive another copy of the survey, or withdraw from the study between 05.09.2022–16.09.2022. Lastly, an SMS text message was sent on 21.09.2022 to people who did not respond to the phone calls with the survey link, an option to receive a hard copy, or withdraw.

### Measures

The phase 1 survey included the following demographic questions: sex at birth, date of birth, ethnicity, relationship status, current employment, number of dependents (individuals who directly depend on the participant for their expenses), and highest educational attainment.

For the purposes of this feasibility study, we specifically included screening measures for the 12 categories of mental disorders as classified by the DSM-5. We included this wide breadth of screening measures to identify which questionnaires to include in the population-based survey and to identify which measures are systematically missed by respondents. The following screening measures were included:

1. Prodromal Questionnaire - 16 Item Version (PQ-16) for schizophrenia spectrum and other psychotic disorders [13];
2. Mood Disorder Questionnaire (MDQ) for bipolar and related disorders [14];
3. Patient Health Questionnaire- 9 (PHQ-9) for depressive disorders [15];
4. Generalised Anxiety Disorder Questionnaire (GAD-7) for anxiety disorders [16];
5. Zohar-Fineberg Obsessive Compulsive Screen (Z-FOCS) for obsessive–compulsive disorder (OCD) [17];
6. Post-Traumatic Stress Disorder Checklist for DSM-5 (PCL-5) for post-traumatic stress disorder (PTSD) [18];
7. Diabetes Eating Problems Survey -Revised (DEPS-R) for eating disorders [19];
8. Pittsburgh Sleep Quality Index (PSQI) for sleep disturbances [20];
9. Female Sexual Function Index- 6 (FSFI-6) or the Brief Male Sexual Function Inventory (BSFI) for sexual dysfunctions [21, 22];
10. Alcohol Use Disorders Identification Test (AUDIT) [23];
11. Drug Abuse Screen Test-10 (DAST-10) for substance-related and addictive disorders [24];
12. Standardised Assessment of Personality – Abbreviated Scale (SAPAS) for personality disorders [25].

In addition, the Type 1 Diabetes Distress Scale (T1-DDS) for diabetes-related distress [26], Confidence in Diabetes Self-Care (CIDS) for diabetes-specific self-efficacy [27] and Multidimensional Scale of Perceived Social Support (MSPSS) for perceived social support [28] were included. Higher scores on all measures indicate a higher likelihood of clinical impairment and/or distress. All measures were included following consultations with the Wessex Diabetes Patient Public Involvement and Engagement (PPIE) group.

### Phase 2: clinical interview

For those interested in phase 2, an interview was arranged either face-to-face or online at times convenient for participants including out of hours. The enhanced modules of the Structured Clinical Interview for DSM-5, Researcher Version (SCID-5-RV) [29] were administered for attention-deficit/hyperactivity disorder (ADHD), schizophrenia spectrum and other psychotic disorders, bipolar and related disorders, depressive disorders, anxiety disorders, obsessive–compulsive and related disorders, trauma and stressor-related disorders, somatic symptom and related disorders, feeding and eating disorders, sleep–wake disorders, disruptive, impulse-control, and conduct disorders and substance-related and addictive disorders.

### Biomedical assessment

Medical records collected from participants’ GP practice included their mean HbA1c in the past year, most recent HbA1c, age at T1D diagnosis, diabetes duration, number of admissions for severe hypoglycaemia and DKA, and insulin treatment modality.

### Feasibility and acceptability assessment

We aimed to assess feasibility outcomes at each phase of the study. At phase 1 the feasibility outcomes were the proportion of participants who were a) identified as eligible from the GP register; b) invited to the study; c) eligible for phase 1 survey and d) completed the phase 1 survey. At phase 2 the feasibility outcomes were the proportion of people who a) consented to a follow-up for phase 2; b) lost to phase 2 follow-up after consenting; c) participated in the phase 2 interview. Feasibility outcomes were to also describe the sociodemographic characteristics of responders versus non-responders at phases 1 and 2, and whether there were any associations between mental disorders, diabetes-related functioning, and the biomedical status in responders versus non-responders. We also calculated Cohen’s kappa statistic used to assess concordance between case-ness at phases 1 and 2.

Additionally, we aimed to assess the acceptability of a) the survey content, its delivery and participation burden and b) the recruitment strategy and process from the GP surgery through an optional open-ended free text question asking for feedback on the survey or study as a whole. We analysed the responses by scoring the frequency of comments. We did not pre-specify progression criteria to a larger study as this was an exploratory study to understand patterns of response and identify barriers to uptake which would be addressed to optimise recruitment in future studies.

### Ethical considerations

Ethics approval was obtained from the National Health Service (NHS)—Health Research Authority (Reference: 21/NE/0177).

### Statistical analysis

Proportions (with 95% confidence intervals (CI)) were used to estimate the feasibility parameters. Quantitative data to assess acceptability from the participant and GP perspective were imported to the Statistical Package for Social Sciences (SPSS) Version 28.0.1.1 [30] software for analysis and reported descriptively and graphically. To account for the different types of missing data which might vary by measure, and to systematically asses acceptability in a standardised manner, a measure was considered as complete (not missing) if the value of the numerator was greater than or equal to the cut-off score (denominator) for case-ness even if some of the questions had missing values in that measure [31]. We then determined that if 30% of the participants at phase 1 did not complete a measure (missing), that measure was considered unacceptable [32].

## Results

### Feasibility outcomes

There were 146 adult patients with T1D registered at the four GP surgeries. Of the 105 eligible participants, 52.4% [95% CI: 42.4–62.2) (*n* = 55) completed the phase 1 survey, of which 45.5% [95% CI: 37.19–53.81] (*n* = 25/55) completed the phase 2 diagnostic interview. The demographic characteristics of responders in phases 1 and 2 are summarised in Table 1, and the study flow reporting the feasibility outcomes is illustrated in Supplemental File 1.

**Table 1:** Demographic characteristics of responders at phases 1 and 2

Characteristics	Phase 1 (*n* = 55)	Phase 2 (*n* = 25)
	mean (SD)	mean (SD)
Age (years), range 20 to 79 years	57.2 (16.9)	56.8 (16.6)
*n* (%)		
Sex (female)	28 (50.9)	15 (60.0)
Ethnicity		
White	55 (100)	25 (100.0)
Black or minority ethnicity	0 (0.0)	0 (0.0)
Missing	0 (0.0)	0 (0.0)
Relationship status		
Single	13 (23.6)	7 (28.0)
Married/cohabiting	41 (74.5)	18 (72.0)
Missing	1 (1.8)	0 (0.0)
Employment status		
Student	4 (7.3)	2 (8.0)
Employed	19 (34.5)	8 (32.0)
Unemployed/retired	30 (54.5)	13 (52.0)
Missing	2 (3.6)	2 (8.0)
Number of dependents		
None	35 (63.6)	18 (72.0)
1 dependent	6 (10.9)	3 (12.0)
2–4 dependents	12 (21.8)	4 (16.0)
Missing	2 (3.6)	0 (0.0)
Highest educational attainment		
GCSEs or equivalent	16 (29.1)	8 (32.0)
A-levels or equivalent	16 (29.1)	8 (32.0)
University or higher qualification	19 (34.5)	9 (36.0)
Missing	4 (7.3)	0 (0.0)

The medical records of responders and non-responders are reported in Table 2. In phase 1, 27.3% (*n* = 15/55) of responders had a documented lifetime history of at least one mental disorder, compared to 54.0% (*n* = 27/50) of non-responders. At phase 1, depressive disorders were the most recorded diagnosis in responders (66.7%, *n* = 10/15) and non-responders (88.9%, *n* = 24/27). Of the responders with depressive disorders, comorbidities included anxiety disorders, trauma- and stressor-related disorders and substance-related and addictive disorders, each at 6.7% (*n* = 1/15). Additional diagnoses among responders included anxiety disorders without comorbid depression (20.0%, *n* = 3/15) and engagement in psychotherapy without a formal mental disorder diagnosis (6.7%, *n* = 1/15). Phase 1 non-responders had a record of a broader range of comorbid mental disorders, namely neurodevelopmental disorders (14.8%, *n* = 4/27), anxiety disorders (11.1%, *n* = 3/27), trauma- and stressor-related disorders (3.7%, *n* = 1/27), feeding and eating disorders (3.7%, *n* = 1/27), substance-related and addictive disorders (7.4%, *n* = 2/27), neurocognitive disorders (3.7%, *n* = 1/27), and personality disorders (7.4%, *n* = 2/27). A further subset had a record of neurodevelopmental disorder (3.7%, *n* = 1/27), trauma- and stressor-related disorder (3.7%, *n* = 1/27), or a mental disorder not otherwise specified (3.7%, *n* = 1/27) without comorbidities. Those who responded to phase 1 had lower HbA1c values, only two had had 1 severe hypoglycaemic episode, two had had 2 severe hypoglycaemic episodes and five had had 1 DKA episode. Phase 1 responders were also diagnosed at an older age, had a longer duration of T1D than non-responders whom four had had 1 severe hypoglycaemic episode and two had had 3 severe hypoglycaemic episodes, and five had had 1 DKA episode, two had had 2 DKA episodes.

**Table 2:** Assessment of participation bias by diabetes status at phases 1 and 2

Characteristics	Phase 1		Phase 2	
	Responders (*n* = 55)	Non-responders (*n* = 50)	Responders (*n* = 25)	Non-responders (*n* = 80)
	mean (SD)			
Recorded mental disorder in lifetime	15 (27.3)	27 (54.0)	8 (32.0)	21 (26.3)
HbA1c in the past year, mmol/mol	63 (14.2)	73 (21.0)	62 (12.2)	69 (19.4)
Most recent HbA1c, mmol/mol	60 (12.6)	71 (22.0)	61 (12.5)	66 (21.6)
Age at diabetes diagnosis, years	27 (17.4)	24 (15.4)	26 (15.2)	26 (16.9)
Diabetes duration, years	30 (18.3)	24 (15.5)	30 (16.7)	25 (18.1)
	n (%)			
Insulin treatment modality				
Continuous subcutaneous insulin infusion	34 (61.8)	29 (58.0)	17 (68.0)	45 (56.3)
Multiple daily self-injections	18 (32.7)	18 (36.0)	7 (28.0)	30 (37.5)
Missing	3 (5.5)	3 (6.0)	1 (4.0)	5 (6.3)

In phase 2, 32.0% (*n* = 8/25) of responders had a record of at least one lifetime mental disorder, compared to 26.3% (*n* = 21/80) of non-responders. Of the responders with mental disorder diagnoses, depressive disorders were again most common at 62.5% (*n* = 5/8), followed by anxiety disorders (37.5%, *n* = 3/8). Non-responders had a record of a broader range of mental disorders, namely neurodevelopmental disorders (1.3%, *n* = 1/80), anxiety disorders (2.5%, *n* = 2/80), and combined anxiety and depressive disorders (2.5%, *n* = 2/80). Additionally, 1.3% (*n* = 1/80) had co-occurring anxiety, depressive, and eating disorders. Depressive disorders alone were recorded in 13.8% (*n* = 11/80), trauma- and stressor-related disorders were recorded in 1.3% (*n* = 1/80), and PTSD comorbid with depressive disorder in another 1.3% (*n* = 1/80). Responders in phase 2 also had lower HbA1c values than non-responders, two had had 1 severe hypoglycaemic episode, one had 2 severe hypoglycaemic episodes, and one had had 1 severe hypoglycaemic episode. Summary scores for self-reported survey measures at phase 1 and clinical interview at phase 2 are reported in in Tables 3 and 4.

**Table 3:** Summary description of measures collected at phase 1

Measures, total *n* = 55	mean (SD)	Case-ness, n (%)
Schizophrenia spectrum and other psychotic disorder symptoms		
PQ-16 score	2.0 (2.4)	9 (16.4)
Missing	6 (10.9)	
Bipolar disorder symptoms		
MDQ score	3.0 (2.5)	16 (29.1)
Missing	12 (21.8)	
Depressive disorder symptoms		
PHQ-9 score	6.2 (6.6)	13 (23.6)
Missing	2 (3.6)	
Anxiety disorder symptoms		
GAD-7 score	4.2 (4.6)	7 (12.7)
Missing	1 (1.8)	
Obsessive–compulsive disorder symptoms		
Z-FOCS score	1.0 (1.2)	28 (50.9)
Missing	2 (3.6)	
Post-traumatic stress disorder symptoms		
PCL-5 score	11.8 (13.1)	6 (10.9)
Missing	2 (3.6)	
Eating disorder symptoms		
DEPS-R score	10.5 (9.2)	6 (10.9)
Missing	4 (7.3)	
Sleep disturbance symptoms^a^		
PSQI score	167 (5.5)	
Missing	38 (69.1)	
Female sexual dysfunction symptoms (***n*** = 28)		
FSFI-6 score	2.4 (2.3)	8 (28.6)
Missing	9 (33.3)	
Male sexual dysfunction symptoms (***n*** = 27)^a^		
BSFI score	22.7 (12.1)	
Missing	9 (32.1)	
Alcohol use disorder symptoms		
AUDIT score	5.6 (4.6)	12 (21.8)
Missing	3 (5.5)	
Substance use disorder symptoms		
DAST-10 score	0.4 (1.5)	6 (10.9)
Missing	13 (23.6)	
Personality disorder symptoms		
SAPAS score	3.0 (1.8)	20 (36.4)
Missing	23 (41.8)	
Diabetes-related distress		
T1-DDS score	1.8 (0.7)	3 (5.5)
Missing	8 (14.5)	
Diabetes-specific self-efficacy^a^		
CIDS score	82.8 (15.6)	
Missing	12 (21.8)	
Perceived social support^a^		
MSPSS score	65.3 (16.4)	
Missing	5 (9.1)	

*PQ-16* 16-item Version of the Prodromal Questionnaire, cut-off score (≥ 6), *MDQ* Mood Disorder Questionnaire, cut-off score (≥ 7), *PHQ-9* Patient Health Questionnaire (9-item scale), Cut-off score (≥ 10), *GAD-7* Generalised Anxiety Disorder (7-item scale), Cut-off score (≥ 10), *Z-FOCS* Zohar-Fineberg Obsessive Compulsive Screen, Cut-off score (≥ 1), *PCL-5* PTSD Checklist for DSM-5, Cut-off score (≥ 31), *DEPS-R* Diabetes Eating Problem Survey Revised, Cut-off score (≥ 20), *PSQI* Pittsburgh Sleep Quality Index, *FSFI-6* Female Sexual Function Index-6, (≥ 19), *BSFI* Brief Male Sexual Function Inventory, *AUDIT* Alcohol Use Disorders Identification Test, Cut-off score (≥ 8), *DAST-10* Drug Screening Questionnaire, Cut-off score (≥ 3), *SAPAS* Standardised Assessment of Personality – Abbreviated Scale, cut-off score (≥ 3), *T1-DDS* Type 1 Diabetes Distress Scale, Cut-off score (≥ 3), *CIDS* Confidence in Diabetes Self-Care, *MSPSS* Multidimensional Scale of Perceived Social Support

^a^No validated cut-off scores available

**Table 4:** Percentage of people who had at least one DSM-5 mental disorder using the SCID-5-RV at phase 2 (*n* = 25)

DSM-5 category	n (%)
Attention-deficit hyperactivity disorder	3 (12.0)
Schizophrenia spectrum and other psychotic disorders	1 (4.0)
Bipolar and related disorders	0 (0.0)
Depressive disorders	17 (68.0)
Anxiety disorders	14 (56.0)
Obsessive–compulsive and related disorders	1 (4.0)
Trauma- and stressor related disorders	4 (16.0)
Somatic symptom and related disorders	0 (0.0)
Feeding and eating disorders	5 (20.0)
Sleep–wake disorders	2 (8.0)
Disruptive, impulse-control, and conduct disorders	0 (0.0)
Substance-related and addictive disorders	2 (8.0)

In phase 1, *n* = 13 (23.6%) screened negative for all mental disorders, *n* = 14 (25.5%) screened positive for one mental disorder, and *n* = 28 (50.9%) screened positive for two or more mental disorders.

In phase 2, *n* = 4 (16.0%) did not meet the diagnostic criteria for any mental disorders while *n* = 7 (28.0%) met the diagnostic criteria for one mental disorder, and *n* = 14 (56.0%) met the diagnostic criteria for two or more mental disorders.

### Acceptability outcomes

Regarding the acceptability of the phase 1 survey, responders gave feedback that the phase 1 survey was too long. The mean duration of online completion (*n* = 13/55) was 1 h 54 min. Responders also gave feedback that questions on sexual dysfunction were too personal, and 32.7% (*n* = 18/55) did not respond to this measure but completed other measures in the survey. As for phase 2, the mean duration of the diagnostic interviews lasted 2 h and responders gave feedback that the duration and content of the interview was acceptable.

Table 5 shows the proportion of agreement between case-ness in phases 1 and 2. Schizophrenia spectrum and other psychotic disorders, depressive disorders and substance-related and addictive disorders were significantly concordant across the two phases.

**Table 5:** Concordance of phase 1 survey and phase 2 interview for case-ness (*n* = 25)

Mental disorder	Phase 1-Phase 2 pairs, n (%)	κ	*p*-value
Schizophrenia spectrum and other psychotic disorders			
Concordant	21 (84.0)	0.286	.041*
Not concordant	4 (16.0)		
Bipolar and related disorders			
Concordant	14 (56.0)	0.026	.591
Not concordant	11 (44.0)		
Depressive disorders			
Concordant	17 (68.0)	0.419	.010*
Not concordant	8 (32.0)		
Anxiety disorders			
Concordant	13 (52.0)	0.085	.504
Not concordant	12 (48.0)		
Obsessive–compulsive and related disorders			
Concordant	12 (48.0)	−0.078	.444
Not concordant	13 (52.0)		
Trauma- and stressor-related disorder			
Concordant	21 (84.0)	0.324	.102
Not concordant	4 (16.0)		
Feeding and eating disorders			
Concordant	21 (84.0)	−0.129	.461
Not concordant	4 (16.0)		
Substance-related and addictive disorders			
Concordant	20 (80.0)	0.474	<.001*
Not concordant	5 (20.0)		

^*^p <.05

While feasibility studies are not typically powered for hypothesis testing, we report the *p*-values for the strength of concordance between Phase 1 and Phase 2 diagnostic findings. These should be viewed as exploratory

## Discussion

The primary aim of our study was to assess the feasibility and acceptability of conducting a two-phase survey. We were able to define the study population and invite all those who were eligible, of which over half responded to phase 1, of which just under half responded to phase 2. This was after three mailings (the last one with monetary incentive) plus a telephone call and reminder SMS text message sent by the GP practice at phase 1. As reported in Supplemental File 1, the response rate decreased with each mailing. Mailings with options to complete the survey on paper or online appeared to optimise recruitment. Our recruitment rate for phase 1 was in keeping with the most recent National Health Survey for England and it was borderline acceptable for phase 2. The lower limit of confidence around our estimate in phase 1 was substantially higher than the figure obtained in the National Health Survey which was 32% [33], and lower than two-phase surveys used in occupational cohorts [34] where the participants might have been more likely to respond to due to organisational processes.

We identified four potential barriers to recruitment. First, there were problems identifying the correct address for a quarter of the study population as the surgery was dependent on patients informing them of contact detail changes. Methodology for optimising correct current contact details would need to be considered in the future protocols.

Second, from medical records, we observed differences between responders and non-responders, suggesting participation bias. Non-responders were not only more likely to have a lifetime record of at least one mental disorder, they also had a broader diagnostic range and more frequent comorbidities, particularly with depressive disorders. Responders, in contrast, were biomedically healthier, with lower HbA1c levels and fewer acute diabetes complications. This aligns with previous research linking diabetes complications with poorer mental health outcomes [35–37]. Importantly, even among this comparatively healthier group, over three quarters of phase 1 responders screened positive for at least one mental disorder, and over 80% of phase 2 responders met criteria for a DSM-5 disorder, frequently with comorbidity. Taken together, these findings suggest that mental disorders are common in people with T1D, and that underrepresentation of people with more severe mental disorders or a history of biomedical complications in research could lead to underestimation of prevalence and treatment needs. Future studies would need to consider recruiting from both primary and secondary care (hospital) diabetes services.

Third, although the uptake for phase 1 was acceptable, it may be further improved by reducing the number of screening measures. In the acceptability assessment, participants stated that the number of measures were burdensome, and some found the sexual health questionnaires too personal. Although there is no consensus on what a statistically valid cut-off for the proportion of missing data is [31, 32], the proportion who had missing values for sleep disturbances, sexual dysfunctions and personality disorders were over 30%, suggesting that these screening measures were also not acceptable. Our preliminary findings noted that schizophrenia spectrum and other psychotic disorders, depressive disorders and substance-related and addictive disorders were concordant between phase 1 and 2 pairs. Although feasibility studies are not typically powered for statistical hypothesis testing, we retained *p*-values in this analysis to explore the level of diagnostic agreement across phases. This exploratory approach helps to inform which mental disorders might be prioritised for screening in future streamlined versions phase 1 surveys.

Fourth, the uptake of phase 2 was just under 50%. Over half of the responders to phase 2 were retired and the majority did not have dependents, suggesting that the two hours of interviews may be prohibitive for those who were at work or had caring roles, and anecdotally some of the phase 2 non-responders did report these verbatim as their explanation. Future studies would need to reduce the clinical diagnostic interview duration by tailoring the phase 2 interviews to assess for severe, enduring mental disorders not already reported as current in routine medical records at phase 1 (for instance schizophrenia spectrum and other psychotic disorders, and bipolar disorders), use a shorter research diagnostic interview (such as the Mini-International Neuropsychiatric Interview (M.I.N.I.) [38], and/or offer reasonable honorarium for the participants’ time.

Although our responders were biomedically a healthier group, it is notable that there were still high rates of mental disorders. This is supported by qualitative studies where people with T1D reported feelings of distress and hopelessness despite optimal and sub-optimal T1D management because of the difficulties faced to reach these levels [39]. In focus groups of healthcare professionals, they reported being more aware of diabetes distress in people with optimal management as patients may be ‘burnt out’ and ‘exhausted’ from trying to reach these levels [40]. Additionally, the high rate of comorbidity in our sample suggests that screening for single mental disorders may, again, lead to underestimation of complex mental health problems in T1D.

The discordance between routinely recorded diagnoses in primary care and systematically assessed diagnoses in our two-phase design highlights the limitations of relying on medical records alone. For example, although 54% of phase 1 non-responders had at least one recorded mental disorder, phase 2 diagnostic interviews revealed a higher number of psychiatric multimorbidity than was recorded in primary care for responders. Anxiety disorders, trauma-related disorders, and eating disorders were frequently missed or under-recorded in primary care data. These discrepancies underscore both the fallibility of routine data collection and the need for structured assessment tools when researching mental health in chronic medical populations.

### Strengths and limitations

Strengths included the use of validated questionnaires for mental disorders to help reduce measurement bias. The methods were informed by a multidisciplinary team of physicians, psychiatrists, and people with lived experience. We also used a defined geographical population to avoid selection bias by recruiting from social media platforms. We deliberately deviated from standard two-phase survey design by inviting all the participants who responded to the phase 1 survey to the phase 2 interviews to maximise the number of participants we could collect data from and by including many screening measures rather than a single measure at phase 1 to maximise detection of all psychiatric morbidity and disease specific psychopathology in a medical population. Further, the results of this feasibility study can be used by other researchers and service-providers to inform an appropriately powered definitive survey.

Limitations are that this was a small study which did not allow us to generate prevalence estimates with precision, however, this was not our intention. We only recruited from one small population and the findings in relation to feasibility may not be generalisable to other settings, particularly with regards to people belonging to under-represented ethnic groups as all our sample were White. Selection bias may have been a limitation too, as people were more open to talking about their mental health and T1D may have been more likely to participate, and the non-responders were a more ill group. We did not screen for neurodevelopmental disorders, dissociative disorders, somatic symptom and related disorders, elimination disorders, gender dysphoria, disruptive, impulse-control and conduct disorders, neurocognitive disorders, and paraphilic disorders because we recognise that they are less common, usually already recorded in routine care, and their additions would add to the burden on the participant.

## Conclusions

A two-phase population-based survey design is feasible in terms of participation rates, especially at phase 1, and acceptable for most screening measures. Future studies should consider opting for a minimum questionnaire schedule that includes schizophrenia spectrum and other psychotic disorders, depressive disorders, anxiety disorders and substance-related and addictive disorders in phase 1 to reduce participant burden. Identifying the distribution, comorbidity, and severity of mental disorders in people with T1D and their correlation with severity of biomedical and diabetes complication status are also the next steps in order to identify treatment needs.

## Supplementary Information

Supplementary Material 1.

## Data Availability

All data generated or analysed during this study are included in this published article and its supplementary information files.

## Abbreviations

DSM-5: Diagnostic and Statistical Manual of Mental Disorders - 5th Edition
T1D: Type 1 diabetes
ADHD: Attention-deficit/hyperactivity disorder
AUDIT: Alcohol Use Disorders Identification Test
BSFI: Brief Male Sexual Function Inventory
CI: Confidence interval
CIDS: Confidence in Diabetes Self-Care
DAST-10: Drug Abuse Screen Test-10
DEPS-R: Diabetes Eating Problems Survey -Revised
DKA: Diabetic ketoacidosis
FSFI-6: Female Sexual Function Index- 6
GAD-7: Generalised Anxiety Disorder Questionnaire
GP: General Practitioner
HbA1c: Haemoglobin A1c
M.I.N.I.: Mini-International Neuropsychiatric Interview
MDQ: Mood Disorder Questionnaire
MSPSS: Multidimensional Scale of Perceived Social Support
NHS: National Health Service
PCL-5: Post-Traumatic Stress Disorder Checklist for DSM-5
PCN: Primary Care Network
PHQ-9: Patient Health Questionnaire - 9
PPIE: Patient Public Involvement and Engagement
PQ-16: Prodromal Questionnaire - 16 item version
PSQI: Pittsburgh Sleep Quality Index
SAPAS: Standardised Assessment of Personality – Abbreviated Scale
SCID-5-RV: Structured Clinical Interview for DSM-5, Researcher Version
SPSS: Statistical Package for Social Sciences
T1-DDS: Type 1 Diabetes Distress Scale
Z-FOCS: Zohar-Fineberg Obsessive Compulsive Screen
