# Supplementary material for: Feasibility and acceptability of a two-phase survey for estimating the prevalence of mental disorders in adults with type 1 diabetes
Source: Pilot Feasibility Stud. 2025 Aug 28;11:115. doi: 10.1186/s40814-025-01669-7 (PMC12395789; doi:10.1186/s40814-025-01669-7)
Supplement: Supplementary file 1 — Supplementary Material 1. [file 40814_2025_1669_MOESM1_ESM.docx]

**Supplementary Files**

**Supplemental File 1.**

**Figure 1.** Study flow chart

**Identification**

Identified as potentially eligible from the GP register (n=146)

Deceased n=4

Non-confirmed addresses n=37

Eligible and invited to the study (n=105)

Completed phase 2 interview (n=25)

*(of which video call n=10, in-person n=8, telephone n=7)*

Lost to phase 2 follow-up after consenting (n=9)

**Phase 1**

Responders (n=55)

*(of which paper n=41, online n=13, phone call n=1)*

Mailings and contacts:

1^st^ pack via mail n=24

2^nd^ reminder pack via mail n=19

3^rd^ reminder pack via mail with incentive n=8

4^th^ reminder calls n=4

5^th^ reminder text messages n=0

Consented to follow-up for phase 2 (n=34)

**Phase 2**
